# Genetic analysis of reproductive performance in sows during porcine reproductive and respiratory syndrome (PRRS) and porcine epidemic diarrhea (PED) outbreaks

**DOI:** 10.1186/s40104-019-0330-0

**Published:** 2019-03-01

**Authors:** Cassandra L. Scanlan, Austin M. Putz, Kent A. Gray, Nick V. L. Serão

**Affiliations:** 1 Department of Animal Science, Iowa State University, Ames, IA 50011 USA; 2 Department of Animal Science, North Carolina State University, Raleigh, NC 27607 USA; 3 Genetic Research and Development, Smithfield Premium Genetics, Rose Hill, NC 28458 USA

**Keywords:** Genetic evaluation, Porcine epidemic diarrhea, Porcine reproductive and respiratory syndrome, Reproductive performance, Swine

## Abstract

**Background:**

Porcine reproductive and respiratory syndrome (PRRS) is one of the most infectious swine diseases in the world, resulting in over 600 million dollars of economic loss in the USA alone. More recently, the USA swine industry has been having additional major economic losses due to the spread of porcine epidemic diarrhea (PED). However, information regarding the amount of genetic variation for response to diseases in reproductive sows is still very limited. The objectives of this study were to identify periods of infection with of PRRS virus (PRRSV) and/or PED virus (PEDV), and to estimate the impact their impact on the phenotypic and genetic reproductive performance of commercial sows.

**Results:**

Disease (PRRS or PED) was significant (*P* < 0.05) for all traits analyzed except for total piglets born. Heritability estimates for traits during Clean (without any disease), PRRS, and PED ranged from 0.01 (number of mummies; Clean and PED) to 0.41 (abortion; PED). Genetic correlations between traits within disease statuses ranged from −0.99 (proportion born dead with number weaned; PRRS) to 0.99 (number born dead with born alive; Clean). Within trait, between disease statuses, estimates ranged from − 0.17 (number weaned between PRRS and PED) to 0.99 (abortion between Clean and PRRS).

**Conclusion:**

Results indicate that selection for improved performance during PRRS and PED in commercial sows is possible and would not negatively impact performance in Clean environments.

## Introduction

Porcine reproductive and respiratory syndrome (PRRS) is one of the most infectious swine diseases in the world. Animals infected with the PRRS virus (PRRSV) show respiratory symptoms and impaired performance, such as slower growth rates in newborn and growing pigs and reproductive failure in pregnant sows [1]. This major disease results in 664 million dollars of economic loss per year to the USA swine industry [2].

More recently, another disease that has been causing severe economic impacts in the USA swine industry is porcine epidemic diarrhea (PED). Pigs of all ages infected with the PED virus (PEDV) show diarrhea and vomiting, with affected piglets experiencing nearly 100% mortality within 2 to 3 d of birth [3, 4].

Vaccination and biosecurity have been the main prevention strategies to control PRRS. Although these strategies have shown to limit the impact of this disease at some degree, additional strategies should be evaluated to help further decrease the impact of PRRS. Recent studies have suggested that selection for improved performance in PRRSV-infected sows is possible [5–8]. These authors reported moderate to low heritability estimates for reproductive performance in infected sows. For PED, there is even less information in the literature, with only one genomic study to date, in which they identified regions associated with piglet recovery and death during PEDV infection, but no genetic parameters were estimated [9].

The objectives of this study were: 1) to identify periods of infection of PRRSV and/or PEDV, 2) to estimate the impact of diseases (PRRS and/or PED) on reproductive performance of commercial sows, and 3) to estimate genetic parameters within and between challenged and non-challenged environments.

## Materials and methods

### Data

Performance data and a five-generation pedigree were available from 10 commercial farms in North Carolina, USA. Data included 21,160 farrowing records from 5,352 Large White × Landrace crossbred multiparous sows farrowing from April 2013 to January 2016. All sows used in this study were first parity gilts when collection started, and no new animals were added to the dataset. At the start of data collection, all the sows used were PRRS- and PED-negative for these viruses. The sows used were fully pedigreed and were progeny of 100 sires and 1,595 dams. Progeny of sires were well distributed across farms, with only 8 sires present in 3 or fewer farms. On average, sires had 5.96 progeny sows per farm. Traits analyzed included abortion (AB; a binary trait with either 0 [nonevent] or 1 [event]), total number of piglets born (TB, pigs/litter; calculated as sum of NBA, SB, and MUM), number of piglets born alive (NBA, pigs/litter), number of stillborn piglets (SB, pigs/litter), number of mummified piglets (MUM, pigs/litter), number of piglets born dead (NBD, pigs/litter; calculated as the sum of SB and MUM), proportion of piglets born dead (PROP, pigs/litter; calculated as NBD/TB), and number weaned (NW, pigs/litter). Traits with a large number of zeros (SB, MUM, and NBD) were analyzed as the natural log of the phenotype + 1 in order to create a more normal and narrow distribution for those traits [8, 10]. Sows with duplicated identification (ID) numbers (i.e. wrong duplicated IDs) were removed as well as those with TB greater than 25 or less than 3. After data editing, 20,796 farrow events from 5,314 sows were used for analyses. The number of parities ranged from 1 to 8 with an average parity of 3.0 (SD = 1.7). The average number of animals and farrowing records per farm was 541.2 (SD = 186.0) and 2,195.6 (SD = 741.9), respectively. Table 1 shows summary statistics of the traits analyzed.

**Table 1:** Summary statistics of the raw data

Trait^a^	*n*	Mean	SD	Min	Max
AB, %	20,558	4.06	19.73	–	–
TB	21,197	14.16	3.32	3	25
NBA	20,540	12.86	3.25	0	25
SB	20,540	0.90	1.31	0	15
MUM	20,540	0.39	1.02	0	20
NBD	20,540	1.29	1.75	0	20
PROP	20,540	0.09	0.12	0	1
NW	20,043	9.31	3.67	0	16

^a^AB, Percent of abortions; TB, Total number of piglets born; NBA, Number of piglets born alive; SB, Number of stillborn piglets; MUM, Number of mummified piglets; NBD, Number of piglets born dead; PROP, Proportion of piglets born dead; NW, Number of piglets weaned

### Identification of PRRS and PED outbreaks

Data was split into PRRS and/or PED affected, or Clean status at each farm based on unique herd-year-week (HYW) estimates, as proposed by Rashidi et al. [7]. To obtain HYW estimates for each trait separately, the whole data was analyzed using the following model:

1 $$ {Y}_{ijklm}=\mu +{PAR}_i+{YR}_j+{FARM}_k+{hyw}_l+{sow}_m+{e}_{ijklm} $$

where *Y*_*ijklm*_ is the phenotypic value of a trait; *μ* is the mean; *PAR*_*i*_ is the fixed effect of the *i*^th^ parity; *YR*_*j*_ is the fixed effect of the *j*^th^ year; *FARM*_*k*_ was the fixed effect of the *k*^th^ farm; *hyw*_*l*_ is the random effect of the *l*^th^ herd-year-week, assuming $hyw\sim N\left(0,\boldsymbol{I}{\sigma}_{hyw}^2\right)$, where ***I*** is the identity matrix; *sow*_*m*_ is the random effect of the *m*^th^ sow, assuming $sow\sim N\left(0,\boldsymbol{I}{\sigma}_{sow}^2\right)$; and *e*_*ijklm*_ is the random residual associated with *Y*_*ijklm*_, assuming $e\sim N\left(0,\boldsymbol{I}{\sigma}_e^2\right)$. All traits were analyzed with a linear mixed model, with the exception of AB, in which a logit mixed model was used. A total of 1,332 HYW levels were generated, ranging from 5 to 75 farrowing records per HYW level, with an average of 17.2 (SD =9.13). Because of removal of animals due to standard production procedures, such as lameness, poor insemination rates, and more, there were more data at the beginning of the study, and these decreased as time went on and animals were culled.

Outbreaks of PRRS were identified using only the traits AB, NBA, and NBD, whereas NW was used to identify PED outbreaks. These traits were chosen because an increase in AB and NBD and a decrease in NBA are indicative of a PRRS outbreak [1], and a decrease in NW is indicative of a PED outbreak [4]. HYW estimates were standardized and considered extreme when greater or lower than 1.96 and −1.96, respectively. These values were chosen as they represent the limits for 95% of the data; since specific directions are expected (e.g. decrease in NBA for PRRS outbreaks), a one-tail limit was used. A window of time was deemed as a PRRS outbreak when simultaneous increases in AB and NBD occurred along with a decrease in NBA for a period of two or more consecutive weeks. A decrease in NW for a period of two or more weeks was used to identify PED outbreaks. This strategy to identify PRRS outbreaks was used and shown to be effective by Lewis et al. and Rashidi et al. [7, 10]. Weeks where these traits did not show extreme standardized HYW estimates were considered to be disease free (i.e. Clean). All outbreaks were confirmed with results from periodic serological tests that each farm performed, following their standard operation procedures, in which PRRS and PED outbreaks were confirmed via ELISA and qPCR, respectively.

**Fig. 1:**
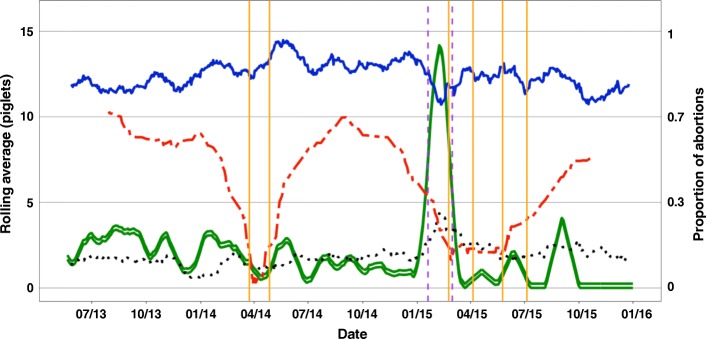
Example of visualization of the performance data using rolling averages (RA) across time (month and year) for one of the farms used in the study. Traits included for visualization were: abortions (AB; green open line), number born alive (NBA; blue solid line), number born dead (NBD; black dotted line) and number weaned (NW; red dashed line). The primary y-axis represents the RA for NBA, NBD, and NW and the secondary y-axis represent the RA for proportion of AB. A 30-day RA was used to visualize all traits. RAs allowed to capture changes in performance due to infection with Porcine Epidemic Diarrhea (PED) or Porcine Reproductive and Respiratory Syndrome (PRRS). Decreases in NW indicated PED whereas PRRS was identified with increases in AB and NBD, and with decreases in NBA. Consecutive vertical lines of the same color represent the initial disease windows that were identified: PRRS (purple dashed line) and PED (orange solid line)

Figure 1 shows predicted disease windows for a single farm atop the rolling averages (RA) for AB, NBA, NBD, and NW. A 30-day RA was used for NBA, NBD and NW, and a 30-day RA of the proportion of abortions was used to depict AB. For AB, the proportion of abortions was defined as the RA of the ratio of the RA of number of abortions events to total events (sum of RA of abortions and RA of farrowing events per day). There were two instances in this data where there was an overlap in the predicted PRRS and PED windows. The overlaps were in total 3 weeks long and contained only 61 records. Preliminary analysis indicated that the mean performance of animals within the overlaps was different than both PRRS and PED, but because of this single event and small sample size, these data were excluded from the analysis.

### Further refining disease statuses

Initial analyses identified 8 and 15 periods of PRRS and PED outbreaks, respectively. The average length (in weeks) of the Clean, PRRS, and PED time windows were 44.1 (SD = 34.3), 5.4 (SD = 3.3), and 5.8 (SD = 2.3), respectively, with an average of 637.4 (SD = 652.4), 104.4 (SD = 56.2), and 111.1 (SD = 95.6) farrowing records per time window. However, preliminary analysis (genetic parameters) of the data indicated that the low number of observations per period, particularly for PRRS, resulted in problems with convergence of the model.

In order to fit the PRRS data better, weeks were either added or subtracted from the beginning and the end of the initially predicted time windows. The creation of these new time windows involved systematically adding or subtracting all possible combinations of weeks from − 2 (i.e. removing 2 weeks) to 6 (i.e. adding 6 weeks) on both the beginning of the predicted window and also at the end of the predicted windows. These different combinations were tested for each of the traits to determine which window fit the data best. In addition to potentially increasing the number of records defined as PRRS status, this strategy allowed traits to have different periods of time for PRRS. In other words, PRRS windows were allowed to encompass different time points, depending on the trait, which is biologically reasonable since PRRS will have different effects on a trait depending on the stage of pregnancy at infection, with, for example, SB being expressed before MUM, as the former is due to infection at later stages of gestations, whereas the former at earlier stages [8]. Selection of the new time windows of PRRS (and thus Clean status) was based on several criteria. First, we selected time windows in which the additive genetic variance during the disease was greater than for the Clean status [6]. Akaike information criterion (AIC) was adjusted for the number of data points included in the windows [11] and this was used to choose the final window of time for each trait and disease status, which were then used for all the remaining statistical analyses. The summary for the final time windows is shown in Table 2.

**Table 2:** Summary statistics for time windows during the different disease statuses^a^

Trait^b^	Clean (windows = 31)			PRRS (windows = 8)			PED (windows = 15)		
	Length (SD)	Records	Mean (SD)	Length (SD)	Records	Mean (SD)	Length (SD)	Records	Mean (SD)
AB, %	36.8 (30.3)	18,564	2.8 (16.5)	5.8 (2.5)	309	24.9 (43.3)	5.9 (2.3)	1685	5.7 (23.1)
TB	37.4 (30.4)	18,653	14.2 (3.3)	10.6 (3.1)	970	14.7 (3.6)	5.9 (2.3)	1574	14.0 (3.2)
NBA	38.2 (30.7)	17,708	12.9 (3.2)	12.4 (4.3)	1258	12.0 (3.8)	5.9 (2.3)	1574	12.7 (3.1)
SB	38.4 (30.9)	18,190	0.5 (0.5)	7.0 (4.1)	776	0.7 (0.6)	5.9 (2.3)	1574	0.4 (0.5)
Trait									
MUM	36.7 (30.8)	18,307	0.2 (0.4)	8.1 (2.9)	659	0.4 (0.6)	5.9 (2.3)	1574	0.2 (0.5)
NBD	37.8 (30.8)	18,307	0.6 (0.6)	8.1 (2.9)	659	0.9 (0.8)	5.9 (2.3)	1574	0.6 (0.6)
PROP	36.9 (31.1)	17,974	0.1 (0.1)	9.7 (4.0)	992	0.1 (0.2)	5.9 (2.3)	1574	0.1 (0.1)
NW	36.8 (30.3)	17,732	9.9 (3.1)	11.0 (4.6)	751	8.0 (3.7)	5.9 (2.3)	1560	3.9 (4.7)

Window, number of outbreak windows identified; Length, average length (weeks) of individual outbreak windows; Records, number of records analyzed; Mean, raw means of the records within each trait for the disease windows

^a^Clean, Clean status (no presence of PRRS and PED); PRRS, porcine reproductive and respiratory syndrome; PED, porcine epidemic diarrhea

^b^AB, Abortion; TB, Total number of piglets born; NBA, Number of piglets born alive; SB, Number of stillborn piglets; MUM, Number of mummified piglets; NBD, Number of piglets born dead; PROP, Proportion of piglets born dead; NW, Number of piglets weaned

### Impact of disease on reproductive performance

The impact of the disease statuses (Clean, PRRS, or PED) on reproductive performance was assessed using a two-step approach because of confounding of disease status with other fixed effects in the model. First, reproductive performance data was analyzed with the following model:

2 $$ {Y}_{ijklm}=\mu +{PAR}_i+{YR}_j+{FARM}_k+{u}_m+{pe}_m+{e}_{ijklm} $$

where *Y*, *μ*, *PAR*, *YR*, and *FARM* are as defined previously; *u*_*m*_ is the additive genetic effect of the *m*^*th*^ animal, assuming $u\sim N\left(0,\boldsymbol{A}{\sigma}_u^2\right)$; where ***A*** is the additive relationship matrix; and *pe*_*m*_ is the random effect of the permanent environment on sow *m*, assuming $pe\sim N\left(0,\boldsymbol{I}{\sigma}_{pe}^2\right)$. The ***A*** matrix was estimated using a pedigree of 10985 animals. Second, phenotypes were pre-adjusted (Y*) for the fixed effects of parity, year, and farm, and then the impact of disease status was evaluated using the following model:

3 $$ {Y}_{ij}^{\ast }=\mu +{STAT}_i+{u}_j+{pe}_j+{e}_{ij} $$

where *μ, u,* and *pe* are as defined previously; ${Y}_{ij}^{\ast }$is the adjusted phenotypic value of a trait; *STAT*_*i*_ is the fixed effect of the *i*^th^ disease status. Least-squares means of STAT were estimated and then reconstructed based on the estimates of fixed effects from Eq. 2, according to the proportion of each respective level of *STAT*.

Additionally, the effect of season was also explored in initial analyses. Season was explored as a fixed effect in a number of ways, by month, by time of year (i.e. spring, summer, etc.), and as a seasonality covariate [7]. The effect of season was confounded with disease status as PRRS tends to break during the winter months [12] and was found to be not significant (*P* > 0.1) for this dataset.

### Genetic parameters of reproductive performance during clean and diseased statuses

Genetic parameters (heritability and correlations) were estimated considering each trait defined within disease status (e.g. NBA during PRRS) as a separate trait. The univariate animal model below was used to estimate heritabilities:

4 $$ {Y}_{ijklm}=\mu +{PAR}_i+{YR}_j+{FARM}_k+{RA}_l+{u}_m+{e}_{ijklm} $$

where *Y*, *μ*, *PAR*, *YR*, *FARM*, and *u* are as defined previously; and *RA*_*l*_ is the fixed effect covariate of the RA of the traits analyzed. The effect of RA was fitted in order to account for the average productivity of the farm at a given time, intended to capture the epidemic severity and dynamics of the diseases [8, 11]. For analysis of traits in the Clean status, a random permanent environment (*pe*) effect was added to the model, assuming $pe\sim N\left(0,\boldsymbol{I}{\sigma}_{pe}^2\right)$, in order to account for repeated records (parities) in the same animal. A permanent environmental effect was not fit for PRRS or PED because there were no sows with repeated records for these diseases.

Genetic and phenotypic correlations were estimated using the same models describe above, but in a bivariate fashion. For AB, heritability was estimated using a logit function, but due to convergence problems, genetic correlations were estimated fitting AB as a quantitative variable in a linear mixed model. Correlations were estimated, within disease status, for the same trait, and, within trait, between disease statuses. When analyzing the same traits between disease statuses, it was assumed that there was no residual covariance between them. Similarly, animals that aborted did not have information for other reproductive traits, and the residual (and therefore phenotypic) covariances were not estimable. All statistical analyses were performed in ASReml4 [13].

## Results

### Reproductive performance between diseased statuses

The effect of disease status on reproductive performance can be found in Table 3. Disease status was found to be statistically significant (*P* < 0.05) for all traits, except TB (*P* = 0.68), as expected. In general, Clean and PED had similar reproductive performance, and PRRS had lower performance than both. All levels of status (Clean, PED, and PRRS) significantly (*P* < 0.01) affected outcomes for AB, with 2.9 ± 0.2%, 38.8 ± 0.9%, and 1.6 ± 0.5% incidence of AB in Clean, PRRS, and PED statuses, respectively. Clean and PED were found to be significantly different (*P* < 0.05) than PRRS for NBD, with 0.81 ± 0.01, 1.32 ± 0.03, and 0.82 ± 0.02 piglets for Clean, PRRS, and PED, respectively. Clean and PED were significantly different (*P* < 0.01) from PRRS for MUM, with 0.20 ± 0.01 and 0.22 ± 0.01 piglets for Clean and PED, respectively, and 0.46 ± 0.02 piglets for PRRS. For MUM, Clean and PED were not found to be significantly different (*P* = 0.24). Clean and PRRS were significantly different (*P* < 0.01) for PROP, with estimates of 0.08 ± 0.01 and 0.13 ± 0.01 piglets, respectively, but there was no difference (*P* = 0.23) between Clean and PED, with PED having an estimate of 0.09 ± 0.01 piglets. All statuses were also found to be significantly different (*P* < 0.01) from each other for NW, with 9.51 ± 0.05 (Clean), 8.34 ± 0.13 (PRRS), and 5.58 ± 0.10 (PED) piglets. There was a significant effect of disease status (*P* = 0.03) for NBA, with lower NBA during PRRS (11.53 ± 0.10), compared to both Clean (12.65 ± 0.06) and PED (12.71 ± 0.10), which were statistically similar (*P* = 0.48). This same pattern was found (*P* < 0.01) for SB, in which PRRS (0.84 ± 0.02) had poorer performance (*P* < 0.01) than both Clean and PED statuses (0.60 ± 0.01 and 0.59 ± 0.02, respectively), while these were statistically the same (*P* = 0.38).

**Table 3:** Least squares means (SE) of traits by disease status^1^

Trait^2^	Disease status			*P*-value
	Clean	PRRS	PED	
AB,%	2.9^b^ (0.2)	38.8^a^ (0.9)	1.6^c^ (0.5)	< 0.01
TB	14.12^a^ (0.07)	14.21^a^ (0.12)	14.14^a^ (0.10)	0.66
NBA	12.65^a^ (0.06)	11.53^b^ (0.10)	12.71^a^ (0.10)	0.03
SB ^3^	0.60^b^ (0.01)	0.84^a^ (0.02)	0.59^b^ (0.02)	< 0.01
MUM ^3^	0.20^b^ (0.01)	0.46^a^ (0.02)	0.22^b^ (0.01)	< 0.01
NBD ^3^	0.81^b^ (0.01)	1.32^a^ (0.03)	0.82^b^ (0.02)	< 0.01
PROP	0.08^b^ (0.01)	0.13^a^ (0.01)	0.09^b^ (0.01)	< 0.01
NW	9.51^a^ (0.05)	8.34^b^ (0.13)	5.58^c^ (0.10)	< 0.01

^a,b,c^ Means lacking the same superscript are different at *P*-value < 0.05

^1^Clean, Clean status (no presence of PRRS or PED); PRRS, porcine reproductive and respiratory syndrome; PED, porcine epidemic diarrhea

^2^AB, Abortion; TB, Total number of piglets born; NBA, Number of piglets born alive; SB, Number of stillborn piglets; MUM, Number of mummified piglets; NBD, Number of piglets born dead; PROP, Proportion of piglets born dead; NW, Number of piglets weaned

^3^Results are back-transformed from natural log + 1

### Genetic parameters within disease status

Genetic parameters for sow performance traits during the Clean, PRRS, and PED statuses are shown in Tables 4, 5, and 6, respectively. Variance components for Clean, PRRS, and PED statuses are shown in Table 7. In general, traits had low heritability across all disease statuses. During the Clean status, TB showed the highest heritability and MUM had the lowest heritability with estimates of 0.11 ± 0.02 and 0.01 ± 0.01, respectively. Genetic correlations ranged from − 0.83 ± 0.35 (between AB and NBA) to 0.99 ± 0.01 (between NBD with SB). Phenotypic correlations for the Clean status ranged from − 0.38 ± 0.01 (between PROP and NBA) and 0.88 ± 0.01 (between PROP and NBD).

**Table 4:** Genetic parameters^a^ for the Clean status^b^

Trait^c^	AB	TB	NBA	SB	MUM	NBD	PROP	NW
AB^d^	0.07 (0.05)	NC	− 0.83 (0.35)	0.02 (0.45)	− 0.51 (0.85)	− 0.08 (0.47)	0.27 (0.44)	− 0.35 (0.53)
TB	–	0.11 (0.02)	0.95 (0.02)	0.47 (0.10)	0.22 (0.23)	0.47 (0.11)	0.29 (0.14)	0.34 (0.15)
NBA	–	0.87 (0.01)	0.09 (0.02)	0.17 (0.13)	−0.08 (0.24)	0.17 (0.14)	−0.02 (0.16)	0.46 (0.15)
SB	–	0.29 (0.01)	−0.10 (0.01)	0.07 (0.01)	0.61 (0.22)	0.99 (0.01)	NC	−0.39 (0.15)
MUM	–	0.19 (0.01)	−0.12 (0.01)	0.15 (0.01)	0.01 (0.01)	0.72 (0.15)	0.75 (0.16)	−0.32 (0.35)
NBD	–	0.33 (0.01)	−0.14 (0.01)	0.86 (0.01)	0.60 (0.01)	0.05 (0.01)	0.99 (0.02)	−0.32 (0.18)
PROP	–	0.09 (0.01)	−0.38 (0.01)	NC	0.60 (0.01)	0.88 (0.01)	0.03 (0.01)	−0.43 (0.20)
NW	–	0.08 (0.01)	0.13 (0.01)	−0.07 (0.01)	−0.04 (0.01)	− 0.07 (0.01)	−0.31 (0.03)	0.02 (0.01)

*NC* Not converged

^a^Estimates of heritability (diagonal), and genetic (above diagonal) and phenotypic (below diagonal) correlations

^b^The Clean status was defined as the period of time when no disease was actively present

^c^AB, Abortion; TB, Total number of piglets born; NBA, Number of piglets born alive; SB, Number of stillborn piglets; MUM, Number of mummified piglets; NBD, Number of piglets born dead; PROP, Proportion of piglets born dead; NW, Number of piglets weaned

^d^AB was treated as a quantitative variable and was assumed to have no residual covariance when estimating correlations between this and other traits

**Table 5:** Genetic parameters^a^ for the PRRS status^b^

Trait^c^	AB	TB	NBA	SB	MUM	NBD	PROP	NW
AB^d^	0.17 (0.11)	− 0.08 (0.20)	− 0.22 (0.20)	0.01 (0.22)	NC	0.66 (0.21)	0.37 (0.28)	− 0.48 (0.24)
TB	–	0.16 (0.08)	0.86 (0.14)	−0.18 (0.37)	NC	−0.61 (0.42)	− 0.36 (0.42)	0.09 (0.41)
NBA	–	0.74 (0.02)	0.14 (0.07)	−0.49 (0.32)	− 0.50 (0.75)	− 0.91 (0.24)	− 0.67 (0.27)	0.33 (0.41)
SB	–	0.29 (0.04)	− 0.23 (0.03)	0.16 (0.10)	0.33 (0.92)	0.94 (0.22)	0.92 (0.39)	−0.68 (0.36)
MUM	–	NC	−0.35 (0.03)	0.24 (0.04)	0.03 (0.05)	NC	NC	−0.61 (1.09)
NBD	–	0.27 (0.04)	−0.40 (0.03)	0.83 (0.01)	NC	0.18 (0.12)	0.85 (0.26)	−0.85 (0.30)
PROP	–	−0.02 (0.04)	−0.63 (0.02)	0.62 (0.02)	NC	0.85 (0.01)	0.09 (0.08)	−0.99 (0.36)
NW	–	0.10 (0.04)	0.33 (0.03)	−0.21 (0.04)	−0.23 (0.04)	− 0.30 (0.04)	−0.31 (0.03)	0.11 (0.09)

*NC* Not converged

^a^Estimates of heritability (diagonal), and genetic (above diagonal) and phenotypic (below diagonal) correlations;

^b^PRRS, porcine reproductive and respiratory syndrome;

^c^AB, Abortion; TB, Total number of piglets born; NBA, Number of piglets born alive; SB, Number of stillborn piglets; MUM, Number of mummified piglets; NBD, Number of piglets born dead; PROP, Proportion of piglets born dead; NW, Number of piglets weaned;

^d^AB was treated as a quantitative variable and was assumed to have no residual covariance when estimating correlations between this and other traits

**Table 6:** Genetic parameters^a^ for the PED status^b^

Trait^c^	AB	TB	NBA	SB	MUM	NBD	PROP	NW
AB^d^	0.41 (0.06)	−0.15 (0.10)	− 0.05 (0.19)	0.02 (0.21)	− 0.14 (0.87)	0.12 (0.42)	NC	0.35 (0.12)
TB	–	0.26 (0.05)	0.95 (0.05)	−0.12 (0.29)	NC	0.17 (0.50)	−0.14 (0.88)	0.26 (0.19)
NBA	–	0.85 (0.01)	0.07 (0.05)	−0.23 (0.45)	0.49 (1.43)	−0.04 (0.77)	−0.20 (1.01)	0.14 (0.28)
SB	–	0.30 (0.03)	−0.09 (0.03)	0.06 (0.04)	−0.85 (1.34)	0.87 (0.36)	NC	−0.07 (0.36)
MUM	–	NC	−0.15 (0.02)	0.14 (0.02)	0.01 (0.03)	−0.35 (1.49)	−0.19 (2.12)	− 0.83 (1.67)
NBD	–	0.35 (0.02)	−0.16 (0.02)	0.81 (0.01)	0.67 (0.01)	0.02 (0.03)	NC	−0.58 (0.81)
PROP	–	0.17 (0.03)	−0.38 (0.02)	NC	0.65 (0.01)	NC	0.03 (0.03)	−0.22 (0.49)
NW	–	0.05 (0.03)	0.08 (0.03)	0.01 (0.03)	−0.02 (0.03)	−0.02 (0.03)	− 0.05 (0.03)	0.15 (0.05)

*NC* Not converge

^a^Estimates of heritability (diagonal), and genetic (above diagonal) and phenotypic (below diagonal) correlations

^b^PED, porcine epidemic diarrhea

^c^AB, Abortion; TB, Total number of piglets born; NBA, Number of piglets born alive; SB, Number of stillborn piglets; MUM, Number of mummified piglets; NBD, Number of piglets born dead; PROP, Proportion of piglets born dead; NW, Number of piglets weaned

^d^AB was treated as a quantitative variable and was assumed to have no residual covariance when estimating correlations between this and other traits

**Table 7:** Variance components^ab^ for the Clean, PRRS, and PED statuses^c^

Trait^d^	Clean		PRRS		PED	
	${\sigma}_u^2$	${\sigma}_e^2$	${\sigma}_u^2$	${\sigma}_e^2$	${\sigma}_u^2$	${\sigma}_e^2$
AB	0.17	3.29	5.34	3.29	14.88	3.29
TB	1.13	8.30	2.09	10.78	2.65	7.56
NBA	0.88	8.28	1.87	11.56	0.75	8.96
SB	0.02	0.24	0.06	0.32	0.015	0.25
MUM	0.001	0.15	> 0.001	0.37	> 0.001	0.19
NBD	0.02	0.32	0.10	0.46	0.005	0.37
PROP	> 0.001	0.01	0.003	0.03	> 0.001	0.03
NW	0.17	6.87	1.29	10.01	1.78	10.14

^a^Estimates of additive genetic (${\sigma}_u^2$) and residual (${\sigma}_e^2$) variances;

^b^Variances expressed as %^2^ on the logistic scale for AB, and as piglets^2^ for all other traits

^c^Clean, no disease actively present, PRRS, porcine reproductive and respiratory syndrome, PED, porcine epidemic diarrhea

^d^AB, Abortion; TB, Total number of piglets born; NBA, Number of piglets born alive; SB, Number of stillborn piglets; MUM, Number of mummified piglets; NBD, Number of piglets born dead; PROP, Proportion of piglets born dead; NW, Number of piglets weaned

For PRRS, the highest and lowest heritability estimates were found for NBD and MUM with 0.18 ± 0.12 and 0.03 ± 0.05, respectively. Genetic correlations ranged from − 0.99 ± 0.36 (between PROP and NW) to 0.94 ± 0.22 (between SB and NBD). The phenotypic correlations for the PRRS status ranged from − 0.63 ± 0.02 (between NBA and PROP) to 0.85 ± 0.01 (between NBD and PROP). Additive genetic and residual variances numerically increased from Clean to PRRS for all traits except MUM, where only the residual variance increased and the additive genetic variances from both statuses were very low.

The highest and lowest heritability estimates during PED were found for AB and MUM with 0.41 ± 0.06 and 0.01 ± 0.03, respectively. Genetic correlations ranged from − 0.58 ± 0.81 (between NBD and NW) to 0.95 ± 0.05 (between TB and NBA). There were high genetic correlations between SB with NBD (0.87 ± 0.36) and PROP (0.85 ± 0.33) and between NBD and PROP (0.90 ± 0.16). The phenotypic correlations during the PED status ranged from − 0.38 ± 0.02 (between PROP and NBA) to 0.88 ± 0.01 (between PROP and NBD). From Clean to PED, there was a numerical increase in additive genetic variance for AB, TB, and NW, and in residual variance for NBA, SB, MUM, NBD, PROP, and NW.

### Genetic parameters between disease status

The within trait estimates of genetic correlations between disease statuses for AB, TB, NBA, SB, NBD, and NW are depicted in Table 8. Estimates of genetic correlations between Clean and PED ranged from 0.10 ± 0.56 (NBD) to 0.99 ± 0.36 (AB). The genetic correlation estimates between Clean and PED for TB, NBA and SB were high, with 0.78 ± 0.09, 0.79 ± 0.14, and 0.96 ± 0.25, respectively. The genetic correlation estimate for NW between Clean and PED was moderate, with a correlation of 0.67 ± 0.12.

**Table 8:** Estimates of genetic correlations (SE) between disease statuses^a^

Trait^b^	Disease status		
	Clean-PED	Clean-PRRS	PED-PRRS
AB	0.99 (0.36)	0.99 (0.63)	0.38 (0.14)
TB	0.78 (0.09)	0.88 (0.08)	0.63 (0.18)
NBA	0.79 (0.14)	0.82 (0.13)	0.92 (0.35)
SB	0.96 (0.25)	0.60 (0.15)	0.68 (0.40)
Trait			
NBD	0.10 (0.56)	0.54 (0.29)	0.62 (0.68)
NW	0.67 (0.12)	0.62 (0.20)	−0.22 (0.26)

^a^Clean, Clean status (no presence of PRRS or PED); PRRS, porcine reproductive and respiratory syndrome; PED, porcine epidemic diarrhea

^b^AB, Abortion; TB, Total number of piglets born; NBA, Number of piglets born alive; SB, Number of stillborn piglets; NBD, Number of piglets born dead; NW, Number of piglets weaned

Genetic correlation estimates between Clean and PRRS were moderate to high, ranging from 0.54 ± 0.29 (NBD) to 0.99 ± 0.73 (AB). For TB and NBA, the estimates between Clean and PRRS were high, with correlations of 0.88 ± 0.08 and 0.82 ± 0.13, respectively. For SB and NW, the estimates were moderate, with correlations of 0.60 ± 0.15 and 0.62 ± 0.20, respectively.

Genetic correlation estimates between PED and PRRS ranged from − 0.22 ± 0.26 (NW) to 0.92 ± 0.35 (NBA). The genetic correlation estimate for AB was low (0.38 ± 0.14), whereas for TB, SB and NBD, these were higher, with estimates of 0.63 ± 0.18, 0.68 ± 0.40, and 0.62 ± 0.68, respectively.

## Discussion

### Detecting PRRS and PED outbreaks

There was a clear decrease in performance on every farm that had PRRS and/or PED outbreaks. These deviations from the normal production in each farm is what allowed us to detect the point at which a farm began to show the impact of the diseases. The reproductive losses, including increases in NBD and AB, and decreases in NBA are indicators for PRRS [1], which is why these were the traits used in detecting PRRS outbreaks. The indicator trait used in detecting PED was NW, because high piglet mortality rate is seen during PEDV infection, although piglets are born uninfected [4]. All but one of the 23 identified PRRS and PED outbreaks were confirmed via periodical serological tests performed at each farm. Although this PRRS outbreak was not confirmed serologically, it was retained since the other identified breaks were confirmed and other studies have shown the validity of this method in the identification of disease [10]. Lewis et al. [10] found that using a threshold method to partition animals into healthy and disease statuses has an advantage over partitioning based on serological results because it is stricter and thus, fewer healthy animals would be included in an outbreak window. However, one PRRS outbreak (based on serological results) was not captured using this method. This could be due to lack of severity of infection, so we were unable to capture it, or a false positive from the serological testing. Despite this, the disease windows that were predicted based on the threshold reproductive data are a better representation of the course of the disease because, due to the stringency in predicting windows, the windows are shorter, representing only the time when reproductive performance was actually impaired and thus, only including animals that farrowed during this impaired performance time.

### Reproductive performance between diseased statuses

In our study, we observed an impact of PRRS on all traits, except TB. A previous study by Lewis et al. [10] also found no significant (*P* = 0.06) difference for TB between PRRS and Clean. Differences in TB were not expected because infection prior to implantation of embryos results in resorption of embryos and the sow returns to estrus, but infection after implantation leads to an increase in MUM for infected fetuses [14], which is included in the calculation of TB. Previous studies showed significant decreases (*P* < 0.01) in NBA from 11.1 to 9.7 [10] and 12.8 to 11.6 [5] between Clean and PRRS statuses, respectively, which is in agreement with what was found in the current study, with a decrease from 12.7 (Clean) to 11.5 (PRRS). These studies also found significant decreases in NW between Clean and PRRS that are in agreement with our study. Lewis et al. [10] found a decrease in NW from 10.10 to 8.83 piglets for Clean and PRRS, respectively and Herrero-Medrano et al. [5] found a decrease from 11.00 to 9.35 piglets (*P* < 0.01), ours also showed a similar decrease from 9.5 to 8.3 piglets for Clean and PRRS, respectively. The significant differences between disease statuses for SB and MUM in the current study, with increases from Clean to PRRS, were in agreement with the results reported by Lewis et al. [10], whom found differences for SB (0.62 to 0.84 for Clean and PRRS, respectively) and MUM (− 0.25 to 0.75 for Clean and PRRS, respectively). For PROP, Serão et al. [8] reported an increase from 0.10 to 0.18, between Clean and PRRS, respectively, which was greater but in line with what was found in this study. A significant increase in AB was also found in the current study from 2.9% in Clean to 38.8% in PRRS. No other reports were found for comparison with these results, but the increase in AB is a well-known indicator of PRRS [1].

For PED, there is little information, with only one study to date that compares reproductive performance between Clean and PEDV infection. Since piglets are not infected when the sow is pregnant like they are during PRRSV infection, it is expected that AB, TB, NBA, SB, MUM, NBD, and PROP would be the same between PED and Clean statuses, but that there would be a significant difference for NW between the two. The NW result from the current study was as expected, with a significant decrease in NW from Clean to PED, from 9.5 to 5.6 piglets. Dastiherdi et al. [15] reported an increase in AB in early gestation after a PED outbreak and the raw data for our study indicate the same, with a higher percentage of AB during PED than in Clean (6.6% and 3.1%, respectively). However, once the data was analyzed, we observed a significant difference between Clean and PED in the opposite direction than expected, with a higher AB found during Clean than PED. There were no significant differences found in the current study for TB, NBA, SB, and NBD between Clean and PED, but differences were significant for NW. Using sow performance data in animals that broke with PED at different stages of gestation, [16] reported contrasting results for AB with an increase in AB from 2.0% in Clean to 2.7% in PED (*P* = 0.05). In agreement with our study, these authors found no difference for NBA between Clean and PED with estimates of 11.3 and 11.2 (*P* = 0.38). These authors also found that both SB and MUM increased (*P* < 0.05) from Clean to PED when sows are infected in early gestation. For later gestational infection, no significant difference between Clean and PED for MUM was found, which is in concurrence with our study. Although Olanratmanee et al. [16] reported an increase (*P* < 0.05) in SB from 4.5% (Clean) to 6.2% (PED), we found no differences between both disease statuses.

To our knowledge, there are no studies comparing differences in reproductive performance between sows infected with PRRSV and PEDV. Due to the differences in the diseases, PRRSV infecting piglets in utero and PEDV not infecting piglets until birth, the expectation would be for that there would be significant difference for AB, birth, and weaning traits. For all traits in this study, except TB, PED and PRRS were shown to be significantly different. Based on the indicator traits for these two diseases, it is not too surprising that they were found to be different. PRRS is known to decrease NBA as well as born dead traits, PED has been shown to cause decreases in NW, and both have been shown to cause increases in AB, although we have observed decreased AB during PED in our data analysis. With an increase in born dead and decrease in NBA that is seen in PRRS, it makes sense that the NW would decrease, but not as much as with PED because the mortality rate for PEDV infected piglets is much higher than for PRRS.

One limitation to this study is that since this is commercial data, we do not know which strains of PRRSV or PEDV were present at the farms. Although we are unaware of the strains, this data is representative of what is present in the overall industry. In addition, we must point out that the performance data used for statistical analysis was used to split the data set into disease statuses, based on the biological impact of these diseases on performance. However, Lewis et al. 0] showed that this strategy was successful in splitting data into Clean and Diseased (i.e. PRRS) statuses and capture the effects of the disease. A similar strategy has been used by others, which further validated the approach by Lewis et al. [5, 7, 8]. Finally, one of the objectives of this study was to estimate the impact of PRRS and PED on reproductive performance of sows, and thus, we were able to do so, providing estimates of the differences in performance.

### Genetic parameters within disease status

Heritabilities for reproductive traits are generally low, which is what was observed in this study. In general, heritabilities were similar to those previously reported in reproductive sows in Clean environments, which have been largely discussed in several studies [5, 8, 10], and thus, we will not focus attention in the absence of diseases. Heritability estimates within the PRRS status were low, but higher than those reported during the Clean status. To our knowledge, there are no reports in the literature that include heritability estimates for AB in PRRSV-challenged animals. Heritability estimates for NBA (0.14 ± 0.07), NBD (0.18 ± 0.12), and NW (0.11 ± 0.09) were within the ranges of estimates reported by Lewis et al. [10], Serão et al. [8], and Herrero-Medrano et al. [5]. The estimate for TB (0.16 ± 0.08) in the current study was comparable to the estimate reported by Lewis et al. [10]. Estimates of heritability for SB during PRRS by Lewis et al. [10] and Serão et al. [8] were lower than the what was estimated in the current study. Overall, heritability estimates during PRRS were higher when compared to the absence of disease (i.e. Clean) which is also observed by Lewis et al. [10] and Serão et al. [8]. Standard errors during the PRRS status were generally large, as compared to the Clean status, but this was expected because the PRRS dataset was much smaller than the Clean dataset in this study. In our study, we observed an increase in both additive genetic and residual variances during PRRS compared to the Clean status, with a proportionally greater increase in the additive genetic variance, which resulted in the higher heritability estimates found in PRRS as compared to Clean (data not shown). The larger additive genetic variances and greater heritability in the PRRS status as compared to the Clean status indicate that the genetic differences between animals are more revealed when a disease is present, differently than in an environment without the occurrence of diseases, such as the nucleus herds [5]. Therefore, selection for improved performance under PRRSV infection must be done during the presence of the disease for animals to fully express their genetic potential.

To our knowledge, there are no studies that reported genetic parameters for reproduction traits in sows infected with PEDV. The heritability estimates during PED were comparable to those found in the Clean status, with the exception of AB, TB, and NW, which were higher during PED than during Clean. This overall similarity with the Clean status was expected; infection with PEDV should not have an impact on reproductive performance in sows, as the disease does not infect piglets in utero, so there should be no decrease in TB or increase in the born dead traits with PEDV infection. Since there should be no impact of PED on TB, it was surprising to find that the heritability of TB during PED was estimated to be 0.26 ± 0.05, which was higher than what was found in Clean (0.11 ± 0.02). The moderate heritability estimate for AB (0.41 ± 0.06) was also surprising, since PED is only known for high mortality in piglets. However, this heritability indicates that there is opportunity to select for improved AB in PEDV-infected pigs, which is in accordance with the phenotypically lower AB during PED compared to Clean and PRRS sows. Less surprising was the heritability that was found for NW during PED (0.15 ± 0.05), which was higher than what was estimated during Clean (0.02 ± 0.01) or PRRS (0.11 ± 0.09). Similar to PRRS, there was an increase in both additive genetic and residual variance, with the increase in additive genetic variance being greater, which resulted in increased heritabilities in this study. It is also important to note that during disease, there would be a decrease in cross fostering to limit the spread of disease. When there is a lot of cross fostering and this information is not accurately recorded, genetic variation for of NW cannot be fully captured accurately because the sow and the piglets in her litter are not necessarily related, but more genetic variance can be captured with the increased relatedness of the litter when there is a decrease in cross fostering and thus an increase in heritability of NW can be seen. Although there was an increase in heritability for AB and NW from Clean to PED, the use of these traits for selection purpose during PEDV infection would be challenging. At both the nucleus and commercial levels, AB can be a challenging trait to collect accurately and there is added difficulty in analysis due its binary nature. At the commercial level, there is a high frequency of cross-fostering and limited records kept on these transfers, making genetic evaluations for NW a challenging task to be performed. An added challenge for identifying animals with variation in NW during PEDV infection is the nearly 100% piglet mortality [3, 4]. Nonetheless, our results indicate traits during PRRSV or PEDV infection are, in general, numerically more heritable than in a clean environment.

Genetic and phenotypic correlations were estimated within each of the disease statuses. For the Clean status, most correlations were low, with high genetic and phenotypic correlations for NBD with SB and PROP, and for MUM with PROP, which makes sense since these traits all measure mortality. The low genetic correlations that were found between traits with NW in this study could be due to the lack of traceable cross-fostering information from these animals, which did not allow us to properly account for the foster dam information in the statistical analysis of the data.

During PRRS, the genetic correlation estimates between traits were in general greater than for those in the Clean status. There were also much larger standard errors estimated during PRRS than in Clean. This must be due to the few records for the PRRS status as compared to the Clean status, as well as to the large variation seen during PRRS as compared to Clean for many of the traits. Some traits had opposing genetic correlations between Clean and PRRS, like NBA with SB and NBD which was negative in PRRS and positive during the Clean environment. This pattern indicates that the relationship between born dead traits with NBA is genetically favorable during PRRS as compared to their relationship during Clean. The relationship with NBA and NBD was also much higher than in Clean. Other traits, like NBD with SB and PROP, had genetic correlations that were similar to those that were estimated during Clean. These results indicate that selection for improved performance recorded during a PRRS outbreak in one trait would result stronger changes in other correlated traits, compared to the Clean status. Therefore, selection under Clean status would differ from that under PRRS status. To our knowledge, there are no reports available in the literature providing correlation estimates within PRRS status.

Within the PED disease status, the genetic correlations between AB with NBD and NW, and between SB with NBD were similar in size and direction to their corresponding phenotypic correlations. The genetic correlations between MUM with SB and NW were larger than their corresponding phenotypic correlations, but were the same directionally. The standard errors for the genetic correlations for between MUM with SB, NBD, NBD, PROP, and NW and between PROP and NBA are extremely high. The high genetic correlation between AB and NBD could be due to the similarity in how these traits express performance (i.e. piglets born dead), although one accounts for the number of dead piglets (NBD) and the other does not (AB). This difference between the two may be reflected in their low phenotypic correlations. Compared to the Clean status, much of the genetic correlations were in opposite directions for PED. Comparisons between Clean and PED show that correlated response to selection in a Clean environment for these traits would be different that the response during PED. Also for PED, the standard errors were much larger than for Clean, probably due to the lower number of animals used for the PED analysis and greater residual variance.

There were problems with convergence for models to estimate the relationship of MUM with PROP, NBD, and TB for PRRS, between PROP with NBD and TB with MUM for PED, and between SB and PROP for the Clean status. Within PRRS and PED, it is possible that these problems could be caused by the low number of animals that are within the disease statuses, and the limited number of animals represented in each farm. It is also problematic to estimate genetic correlations for traits where the heritability is not different than zero, like MUM for all disease statuses and PROP for PED, which could also be contributing to these convergence problems.

### Genetic parameters between disease status

Overall, the moderate to high positive correlations between Clean and PRRS statuses found in this study indicated that the underlying genetic mechanisms of these traits are similar between healthy and PRRSV-infected animals, suggesting that selection for improved performance under a PRRS disease status would not negatively affect performance during a Clean environment. In general, estimates found in this study were similar to those found in independent studies. For NBA, the genetic correlation between Clean and PRRS was 0.82 ± 0.12, which was comparable to the estimate by Rashidi et al. [7], but higher than the estimates reported by Herrero-Medrano et al. [5] and Lewis et al. [10]. Genetic correlation between SB in Clean and PRRS was moderate (0.65 ± 0.15) and comparable to the correlation reported by Lewis et al. [10]. There was also a moderate correlation between Clean and PRRS disease status for NBD, 0.47 ± 0.23, which was comparable to the estimate reported in Lewis et al. [10], but lower than the estimates reported by Rashidi et al. [7] and Herrero-Medrano et al. [5]. Herrero-Medrano et al. [5] reported a genetic correlation for NW between the Clean and PRRS status that is comparable to our estimate of 0.59 ± 0.22. The NW estimate reported by Lewis et al. [10] was much lower than what was estimated in the current study, with a genetic correlation of 0.27 ± 0.25 between Clean and PRRS. Genetic correlations for AB were not reported in other studies, but were found to be high between the Clean and PRRS disease statuses, 0.99 ± 0.30. Genetic correlations for TB between disease statuses were also not reported in other studies, but these were also found to be high between Clean and PRRS, 0.88 ± 0.08. Standard errors for many of these genetic correlations were large, most likely due to animals not having records in both environments. This is especially true for AB, where many animals that aborted were removed from the studied herds before having performance recorded under PRRSV-infection.

The expectation for genetic correlations between Clean and PED was that they would be high, since the reproductive performance was, in general, not significantly different between these statuses, with the exception of NW and AB. To our knowledge, there are currently no studies comparing genetic parameters between Clean and PED. Genetic correlations between Clean and PED disease statuses were positive moderate to high for most traits, with the exception of NBD, which had a low genetic correlation (0.11 ± 0.59). There was a significant difference in NBD between these two statuses, and although we may not understand why PED would show a lower NBD than in Clean, this low genetic correlation corroborates with this finding, indicating that, indeed, NBD between Cleaned and PED statuses are different. Nonetheless, the large SE associated with this estimate makes it hard to properly conclude on their genetic relationship. The high genetic correlations between Clean and PED statues for AB, NBA, and SB may be reasonable since PEDV infects the piglet only after birth, so they are born healthy and mortality is high post-infection [3, 4]. With the high post-natal piglet mortality caused by PEDV, it is encouraging that the genetic correlation between Clean and PED was positive and moderate, suggesting that selection for improved NW during Clean would not have a negative impact on NW during PEDV infection.

The genetic correlation estimates between PRRS and PED were more variable than for the previous comparisons. In addition, the standard errors of these estimates were much larger than for the other disease status comparisons, but this should be due to the low number of animals that had records during both statuses. This might also have contributed with the convergence problems we observed for MUM. An added possible practical problem with this trait could be that this trait may not be properly distinguished during recording of the data among the farms due to different staff and different procedures on the farms. Because of these convergence issues, we also estimated these correlations using a sire model (data not shown). The same convergence issues still occurred, and this analysis resulted in the same overall conclusions, but with estimates with much greater SE. Additionally, we used the sire model to investigate potential non-linear relationships between statuses within a trait (data not shown). Sire estimated breeding values (EBVs) for a given trait between statuses were very linearly correlated, with the exception for AB between PRRS and PED. For this trait, PED sire EBVs tended to plateau at high PRRS sire EBVs. However, this dataset consisted of only 100 sires, and with the large SE of estimates, further studies are needed to better understand the relationship between these diseases at the genetic using a sire model. Nonetheless, positive high genetic correlations between PRRS and PED were found for NBA, SB, NBD, and PROP, indicating that reproductive performance will be reflective of the genetic merit of the individual regardless of whether performance was recorded in PRRS or PED. This is of major importance to the swine industry because of the increased interest in breeding a more robust pig that excels in both the Clean and dirty environments. If selection was done for an increase in performance during PRRS, this would also result in increased performance in PED. Moreover, the very low genetic correlation between PRRS and PED for NW might be due to the major impact that PEDV has on NW, and thus, the genetic control for this trait between the two diseases should be quite different. Nonetheless, these results for NW indicate that genetic improvement for response to one disease would not impact the response the other disease.

## Conclusions

Phenotypic and genetic differences were observed in commercial sows as a function of disease status (PRRS, PED, or Clean) in this study. Mean performance under PRRS was different than for performance recorded in Clean and PED affected environments. In contrast, PED and Clean statuses had more similar phenotypic performance. The greater heritability and additive genetic variance estimates obtained during PRRS and PED statuses compared to Clean indicate that selection for improved reproductive performance under these diseases is possible. The high genetic correlations obtained between PRRS and PED statuses indicate that selection for improved reproductive performance under one disease would also be favorable for the other disease. In addition, genetic correlations between Clean and Diseased environments were overall positive, and thus, the reproductive performance in PRRS and/or PED would also be informative of the animal’s genetic merit during Clean. Overall, our results indicate that there is an opportunity to select for improved reproductive performance during PRRS and PED outbreaks in commercial sows.

## Abbreviations

AB: Abortion
AIC: Akaike information criteria
EBV: Estimated breeding value
ELISA: Enzyme-linked immunosorbent assay
HYW: Herd-year-week
ID: Identification
MUM: Number of piglets mummified
NBA: Number of piglets born alive
NBD: Number of piglets born dead
NW: Number of piglets weaned
PED: Porcine epidemic diarrhea
PEDV: Porcine epidemic diarrhea virus
PROP: Proportion of piglets born dead
PRRS: Porcine reproductive and respiratory syndrome
PRRSV: Porcine reproductive and respiratory virus
qPCR: Quantitative polymerase chain reaction
RA: Rolling average
SB: Number of piglets stillborn
SD: Standard deviation
TB: Total number of piglets born
